# Towards Microorganism-Based Biofuel Cells: The Viability of *Saccharomyces* *cerevisiae* Modified by Multiwalled Carbon Nanotubes †

**DOI:** 10.3390/nano10050954

**Published:** 2020-05-17

**Authors:** Ingrida Bruzaite, Juste Rozene, Inga Morkvenaite-Vilkonciene, Arunas Ramanavicius

**Affiliations:** 1 Department of Chemistry and Bioengineering, Faculty of Fundamental Sciences, Vilnius Gediminas Technical University, 10223 Vilnius, Lithuania; 2 Department of Mechatronics, Robotics, and Digital Manufacturing, Faculty of Mechanics, Vilnius Gediminas Technical University, 03109 Vilnius, Lithuania; juste.rozene@gmail.com (J.R.); inga.morkvenaite-vilkonciene@vgtu.lt (I.M.-V.); 3 Laboratory of Electrochemical Energy Conversion, State Research Institute Centre for Physical Sciences and Technology, 10257 Vilnius, Lithuania; 4 Department of Physical Chemistry, Faculty of Chemistry and Geosciences, Vilnius University, 03225 Vilnius, Lithuania; 5 Laboratory of Nanotechnology, State Research Institute Centre for Physical Sciences and Technology, 02300 Vilnius, Lithuania

**Keywords:** biocompatibility, cell viability, carbon nanotubes, *Saccharomyces cerevisiae*, X-ray diffraction

## Abstract

This research aimed to evaluate the toxic effect of multi-walled carbon nanotubes (MW-CNTs) on yeast cells in order to apply MW-CNTs for possible improvement of the efficiency of microbial biofuel cells. The SEM and XRD analysis suggested that here used MW-CNTs are in the range of 10–25 nm in diameter and their structure was confirmed by Raman spectroscopy. In this study, we evaluated the viability of the yeast *Saccharomyces cerevisiae* cells, affected by MW-CNTs, by cell count, culture optical density and atomic force microscopy. The yeast cells were exposed towards MW-CNTs (of 2, 50, 100 μg/mL concentrations in water-based solution) for 24 h. A mathematical model was applied for the evaluation of relative growth and relative death rates of yeast cells. We calculated that both of the rates are two times higher in the case if yeasts were treated by 50, 100 μg/mL of MW-CNTs containing solution, comparing to that treated by 0 and 2 μg/mL c of MW-CNTs containing solution. It was determined that the MW-CNTs have some observable effect upon the incubation of the yeast cells. The viability of yeast has decreased together with MW-CNTs concentration only after 5 h of the treatment. Therefore, we predict that the MW-CNTs can be applied for the modification of yeast cells in order to improve electrical charge transfer through the yeast cell membrane and/or the cell wall.

## 1. Introduction

Microbial fuel cells (MFCs) are the up-coming bioelectronics devices that can be applied for the generation of electricity and cleaning of polluted water [1,2]. During the metabolic redox process, which occurs in living microorganisms, significant electric charge can be generated [3,4]. However, the generation of such ‘bio-electricity’ is limited by the cell membrane and cell wall, which both significantly reduce the ability of yeast cells to transfer charge generated during metabolic processes. Therefore, there is tremendous demand to increase the electrical conductivity of the cell membrane and/or cell wall. In our previous work, we have modified cell membranes of different microorganisms such as *Aspergillus niger* [5,6,7] and some strains of the yeast cells [2,8,9]. This technique enabled us to increase charge transfer through the cell membrane and/or cell wall because the conducting polymer was mostly formed within the cell membrane and/or between the cell membrane and cell wall. Yeast *Saccharomyces cerevisiae* cells are among the best-studied unicellular eukaryotic organisms [10,11]. Therefore, yeast is one of the best candidates to be applied in the design of microbial fuel cells. However, the charge transfer efficiency through conducting polymer nanoparticles is limited because the conductance of conducting polymers is relatively low, in the range of 10^−4^–10^4^ S/m [12,13,14]. Therefore, other nanomaterials with much higher conductivity are required for this purpose. Good candidates for this are carbon-based nanomaterials such as graphene or carbon nanotubes, which possess excellent conductivity, in the range of 10^2^–10^8^ S/m [15,16].

Carbon nanotubes are widely used in the chemical/biological sensors, electronics, biomedicine [17], and applied in the production of conducting fibers to cover various sports equipment (such as battledores, bowling roll balls), what is possible due to their exclusive mechanical, electrical, thermal, and chemical properties (100 times stronger than steel, best field emission emitters, can maintain the current density of more than 10 nA/cm^2^, thermal conductivity comparable to diamond [14]). Therefore, we predict that one of the ways to achieve the MFC goal of improved charge transfer efficiency is to advance the conductivity of these cellular structures by the modification of the cells’ membrane by carbon nanotubes. However, any possible toxicity of these carbon nano-compounds for the microorganisms is still poorly investigated. According to the information in the reviewed literature, positive and negative effects of carbon nanotubes on living cells can be noticed. Studies conducted over the past years have provided compelling evidence that a variety of nanoparticles, including metal oxides, fullerenes, and carbon nanotubes, can cause toxic effects to different kinds of cells [18,19]. Some published data suggest the stimulation of cellular growth by carbon nanotubes [20]. However, the data of other research groups suggest the increase of their mortality towards some kinds of cells, such as murine bone marrow-derived dendritic cells, 3T3 fibroblasts, bronchial epithelial cells and RAW macrophages, human bronchial epithelial cells line [18,21,22,23]. Yeast *Saccharomyces cerevisiae* cells are widely used in cellular biology studies and molecular biology because of their analogy to the cells of higher eukaryotic organisms, and their life cycle is rather short because they are easily cultivated. However, there is not a lot of data concerning the viability of yeast cells treated by the carbon nanotubes.

Therefore, in this work, we investigated the possible influence of the MW-CNTs on the yeast *Saccharomyces cerevisiae* cells and their populations in vitro in order to apply these MW-CNTs modified cells in the microbial biofuel cells.

## 2. Materials and Methods 

### 2.1. Yeast Strain and Chemicals

*Saccharomyces cerevisiae* 21PMR (MAT leu2 ura3-52) was used throughout the conducted experiments. D-glucose powder, yeast extract, peptone powder, and carbon nanotubes were obtained from Sigma-Aldrich (Steinheim, Germany). Double distilled water has been used throughout the experimental work. All other essential reagents and chemicals used in the study were of analytical grade.

For biofuel cell, dried yeast was purchased from a food supplier “Dr. Oetker Lietuva” (Vilnius, Lithuania). 1 g of YPD-broth was mixed with 20 mL distilled water to get medium with YPD-broth 50 g/L concentration. 500 mg of dried yeast was introduced to prepared suspension. A further culture was grown in shaking incubator at 200 rpm till yeast reaches Logarithmic Phase (20–24 h).

### 2.2. Preparation of MW-CNT Suspension

The MW-CNTs stock suspension of 10 mg/mL was prepared in de-ionized water cleaned by a Multi-purpose water purification system, Adrona Crystal EX (Riga, Latvia), ultrasonicated for 30 min by ultrasound bath Sonorex digitec from Bandelin electronic GmbH&Co.KG (Berlin, Germany) and then before use stored in the dark at +4 °C.

### 2.3. Preparation of Solutions for Biofuel Cell

The phosphate buffer solution was prepared with 0.05 M CH_3_COONa; 0.05 M NaH_2_PO_4_; 0.05 M Na_2_HPO_4_, and 0.1 M KCl mixed in distilled water. Glucose and potassium ferricyanide were prepared in phosphate buffer solution. Before investigations, glucose solutions were allowed to mutarotate overnight. 9,10-phenanthrenequinone (PQ) was dissolved in 97% ethanol. 

### 2.4. Graphite Electrode Preparation

Graphite electrode (ø3 mm) was sanded with paper with three different grinding bead sizes and washed with distilled water and 97% ethanol. In the case if PQ was used, it was immobilized on to the prepared graphite electrode by drying 2 µL drop of water containing 3.75 mM of PQ.

### 2.5. Characterization of Nanoparticles

#### 2.5.1. Scanning Electron Microscopy (SEM)

For scanning electron microscopy, the samples with the carbon nanotubes were dispersed in ethanol using an ultrasound bath; the clumps being disintegrated, a drop of the suspension was put on the carbon conductive tab and dried. Before measurements, the surface of MW-CNTs was coated a layer of Au by evaporation in vacuum using a QUORUM Q150RES device (Quorum Technologies Ltd., Laughton, UK). Afterwards, such samples were assayed by scanning electron microscope (SEM) JSM-7600F from JOEL (Tokyo, Japan) [24]. SEM images of MW-CNTs were obtained at various magnifications.

#### 2.5.2. X-ray Diffraction Analysis (XRD)

X-ray diffraction patterns of the samples with the MW-CNTs were recorded with a DRON 6 diffractometer Cu Kα radiation (Joint Stock Company “Bourevestnik”, Saint Petersburg, Russia) (*λ* = 1.54178 Å) at a scanning speed of 0.05° scan/min from *2θ* = 10–70°. The analysis of experimental XRD spectra of MW-CNTs was performed using Crystallografica software (X-ray line profile fitting program XFIT by A. A. Coelho and R. W. Cheary, Sydney, Australia, 2007) and a PDF database [25,26].

#### 2.5.3. Raman Spectroscopy Measurements

MW-NCTs samples for Raman spectroscopy measurements were prepared on a clean slide glass. The Raman spectra were measured using a confocal Raman spectroscopy system (NTEGRA Spectra, NT-MDT) with 532 nm wavelength laser. The laser beam power was 0.17 mW. Raman spectra were collected with an integration time of 10 s.

### 2.6. Exposure of Yeast Saccharomyces Cerevisiae Cells to MW-CNTs

The yeast *Saccharomyces cerevisiae* cells were incubated with aeration in the liquid yeast extract peptone glucose (YPG) medium containing 1% of yeast extract, 2% of peptone, 3% of glucose, for 24 h at 30 °C. Possible toxic effects of present MW-CNTs (of 2, 50 or 100 μg/mL concentrations) were determined by the atomic force microscopy, cell count (yeast cells stained with methylene blue solution and monitored by light microscope [27], the cells were stained with propidium iodide (PI) and monitored by fluorescence microscope) and culture optical density. 

All experiments were carried out in duplicate or triplicate.

### 2.7. Yeast Cell Viability Assays

*Cells count.* The samples of the yeast cells with and without MW-CNTs suspension were centrifuged, and then the supernatants were decanted. The remaining pellet was re-suspended with phosphate-buffered saline (PBS), pH 7.4. Cell preparations were stained with methylene blue solution and analysed by light microscope 1 h after the incubation with the dyes. Light microscope Olympus CX41 from Olympus (Tokyo, Japan) was used optical image acquisition. 

*Fluorescence microscopy.* After the cultivation of the yeast cells with or without MW-CNT suspension, the cells were collected by centrifugation, washed twice, and re-suspended in PBS. Cell suspensions were transferred to the tubes and were stained with PI. A fluorescence microscope Nikon Eclipse Ci-L from Nikon Instruments Inc. (New York, NY, US) was used for the optical image acquisition.

*Analysis of yeast cells surfaces using an atomic force microscope (AFM)*. Yeast cells surface was characterized by AFM (NTEGRA Spectra system, NT-MDT Inc., Moscow, Russia) in tapping mode using commercial silicon cantilevers with a tip diameter of 10 nm and force constant 1.5 N/m. For the preparation of samples for AFM measurements, the agar-grown cells were transferred onto cover slips (Carl Roth, Germany). We did not use any coating on the slide glass to immobilize the cells or any chemical fixation. All experiments have been done at ambient temperature (20 ± 2 °C). Cells surface roughness analysis was done from the scan field area 10 × 10 µm. Data of measurements were analysed using Nova software.

#### Assessment of Temporal Change of Yeast Population Size

The yeast *Saccharomyces cerevisiae* cells were inoculated in the presence of MW-CNT suspension, which contained 2 μg/mL, 50 μg/mL or 100 μg/mL MW-CNTs. In the control sample, MW-CNTs were absent. The yeast cells population size was monitored on liquid YPG medium by measuring the light absorbance of the samples using YPG medium with the cells using a spectrophotometer Genesys 10S UV-VIS from Thermo Fisher Scientific (Vilnius, Lithuania). Another similar sample containing the yeast cells, to which no MW-CNTs suspension was added, was used as a reference. The inoculated incubation medium was kept for 24 h. The initial optical density (OD) of the culture at 600 nm wavelength was 0.1 ± 0.01, which corresponds to 1–2 × 10^6^ of CFU/mL (CFU—colony-forming units).

For the evaluation of data mathematical model developed by Juska [28] was used for calculations:

(1) $$\{\begin{array}{c} \frac{dx}{dt}=\alpha xz\;exp\left(\varsigma \left(z^{3}-1\right)\right)\\ \frac{dz}{dt}=-\mu \;x \end{array}$$

where *x* is population size; *α* is the relative growth rate; *z* is function of the resources that are used to produce the energy; *μ* is the relative death rate.

### 2.8. Electrochemical Measurements

Electrochemical measurements were performed using a Dropsens potentiostat (Methrohm, Utrecht, The Netherlands), and Dropview software. All experiments were carried out at ambient temperature (at 20 °C), while stirring after additional material mixed in PBS, pH = 6.8, under aerobic conditions. Measurements were performed in three electrodes electrochemical cell, where the graphite electrode was connected as a working electrode, the platinum electrode as a counter electrode and Ag/AgCl electrode as a reference electrode. Cyclic voltammograms were recorded at the scan rate of 0.05 V/s in the range from −0.5 V to 0.8 V. Electrochemical impedance was registered at a frequency range from 50 kHz to 10 mHz, amplitude 10 mV.

Two graphite electrodes with different materials immobilized on the electrode surface were used in biofuel cell: one only with 9,10-phenanthrenequinone (PQ) and second with PQ and MW-CNTs. The biofuel cell was made from two-electrodes: one differently modified graphite, and another—platinum electrode. They were placed in a phosphate buffer solution with 70 mM of glucose, 10 mM K_3_[Fe(CN)_6_], and 0.3 mg/mL yeast. During voltage measurements, the external resistances were plugged in parallel to galvanostat for the assessment of power density of the complete biofuel cell.

## 3. Results

### 3.1. Analysis of the Structure of MW-CNTs

**Figure 1:**
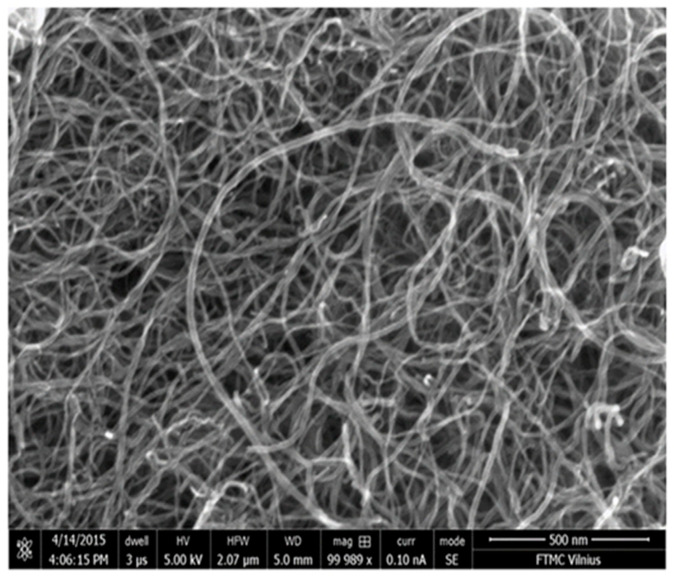
SEM image of carbon nanotube-based nanostructure.

To determine the shape and size of the MW-CNTs used in the present work, the samples were scanned using the scanning electron microscope. The shape of the carbon MW-CNTs was determined by means of the data analysis of SEM measurements. The diameter of MW-CNTs was 10–25 nm, and the length was of several micrometers (Figure 1).

**Figure 2:**
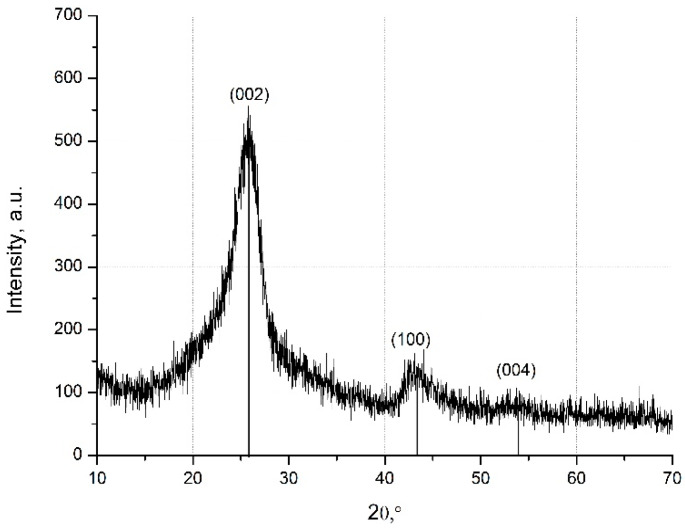
X-ray spectra of the MW-CNTs.

The phase composition of the MW-CNTs was obtained by X-ray diffraction analysis. Three peaks of MW-CNTs (2*θ* = 25.4°; 43.1°; 54°) are present in XRD registered patterns (Figure 2).

The peaks at the angle (*2θ*) of 43.1°and 54° were associated with the (100), (004) diffractions of the hexagonal graphite structure [25]. The most significant diffraction peak at the angle (*2θ*) of 25.4° suggests the typical structure of the multi-layer nanotubes [26,29].

**Figure 3:**
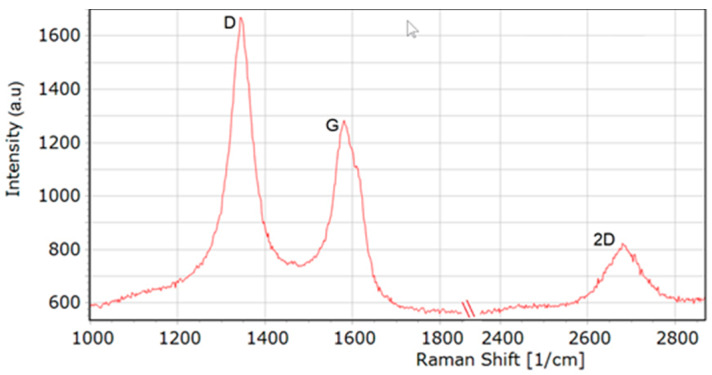
Raman spectra of MW-CNTs used in the experiment.

Figure 3 presents a Raman spectrum of the MW-CNTs used in experiment. A spectrum of MW-CNTs is characterised by the occurrence of the following bands: 1345 cm^−1^ (D band corresponding to the degree of nanotube structural disorder), 1579 cm^−1^ (G band corresponding to the degree of nanotube graphitisation) and 2683 cm^−1^ (2D band corresponding to stresses). The analysis of the shape of D and G modes and the ratio of their ID/IG intensity confirm that we deal with MW-CNTs. Besides, no presence of RBM (Radial Breathing Mode) bands was observed in the investigations performed, which also confirms the use of MW-CNTs.

### 3.2. Evaluation of Toxicity of MW-CNTs on Yeast Cells

**Figure 4:**
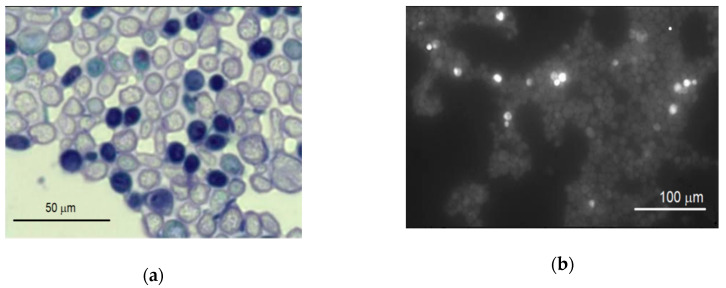
The images of the yeast *Saccharomyces cerevisiae* cells in an identical with field stained by: (**a**) methylene blue (bright cells, living; dark, dead) and (**b**) PI (bright, dead; dark, living).

Initially, the possible effect of carbon nanotubes suspension with various concentrations on the yeast cells was determined by the evaluation of their viability using light and fluorescence microscopes. No difference has been noticed between incubation of either MW-CNT-treated or MW-CNT-untreated (for 24 h) yeast cells. Further, the viability of yeast cells was investigated in more detail. The cells were stained by methylene blue, and both dead cells and the living ones were counted using a light microscope after 1, 2, 3, 20, 22, 24 h from the moment of inoculation. The counts of treated and untreated cells were compared. The dead yeast cells were dyed in blue, as shown in Figure 4a.

**Figure 5:**
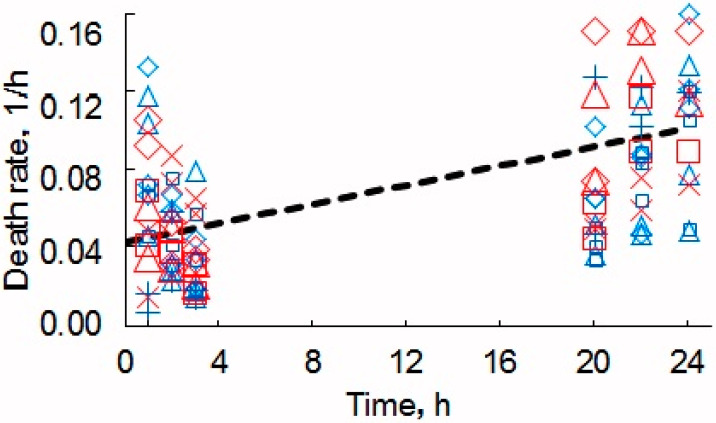
Relative death of the yeast cells. Blue symbols correspond to cell staining with methylene blue and monitoring by optical microscopy, the red, to PI and the fluorescence microscope. The number of cells without MW-CNTs x or + and with MW-CNTs squares, triangles, and rhombs.

The cells were also stained by PI, counted, and compared as previously described using the fluorescence microscope. The results of the staining are presented in Figure 4b. The results of the counts (the ratio of the number of dead cells to that of living ones) are presented in Figure 5.

Finally, a possible effect of the MW-CNTs on the development of the yeast population was investigated. The yeast culture was incubated in the liquid nutritional medium YPD and various concentrations of the carbon nanotubes. The size of the population was estimated spectroscopically. The absorption of the cellular suspension was measured at approximately 600 nm wavelength, both in the control samples (the cells grown in the absence of carbon nanotubes) as well as in the samples with the nanotubes.

**Figure 6:**
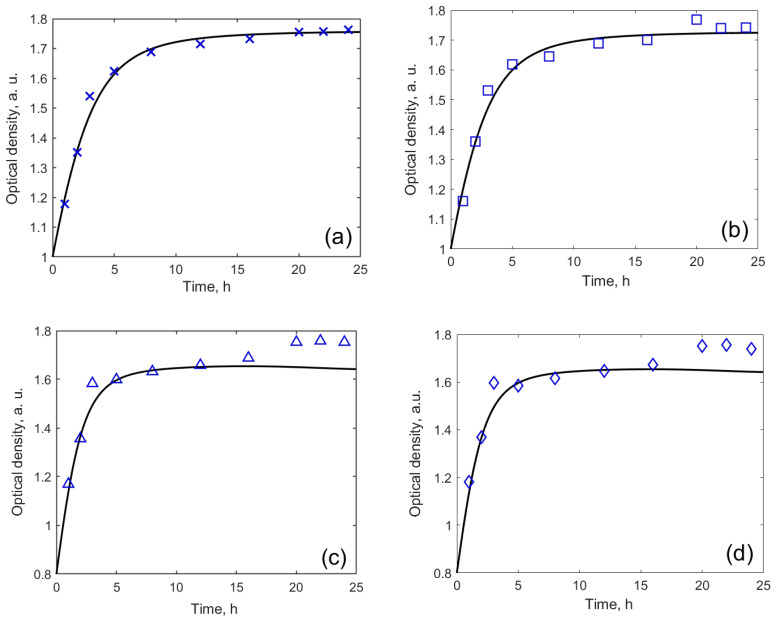
Dependence of optical density on time at different MW-CNTs concentration: (**a**) without MW-CNTs; (**b**) 2 µg/mL MW-CNTs; (**c**) 50 µg/mL MW-CNTs; (**d**) 100 µg/mL MW-CNTs.

Over 24 h, the yeast population is assumed to grow first and then to decline [28,30]. However, our experiment does not show this effect (Figure 6). The size of the population was modelled by equation (1) [28,30]. The model fitted well for MW-CNTs-free medium (Figure 6a) and with those containing 2 µg/mL MW-CNTs (Figure 6b). For 50 µg/mL MW-CNTs (Figure 6c) and 100 µg/mL MW-CNTs, the increase in optical density was observed after 16–24 h. Also, the behaviour of yeast cells treted by solutions containing different concentrations of MW-CNTs is different: in the case when yeasts were treated by solutions containing 0 and 2 µg/mL of MW-CNTs, the experimental results followed the model curve, for the treatment of yeast cells by 50 µg/mL and 100 µg/mL of MW-CNTs containing solutions, from the third-hour results do not changed significantly. Moreover, steady-state conditions were achieved at higher optical density of 1.7 for yeat cells treated by 0 and 2 µg/mL of MW-CNTs, while for other two MW-CNTs concentrations, steady-state conditions were obtained at optical density of 1.6. Relative growth and death rates, calculated by the model, are both two-times higher in the case of treatment by 50 µg/mL and 100 µg/mL MW-CNTs, comparing to other concentrations (Table 1). This suggests, presumably, some effect of the MW-CNTs on the development of the yeast cell population.

**Figure 7:**
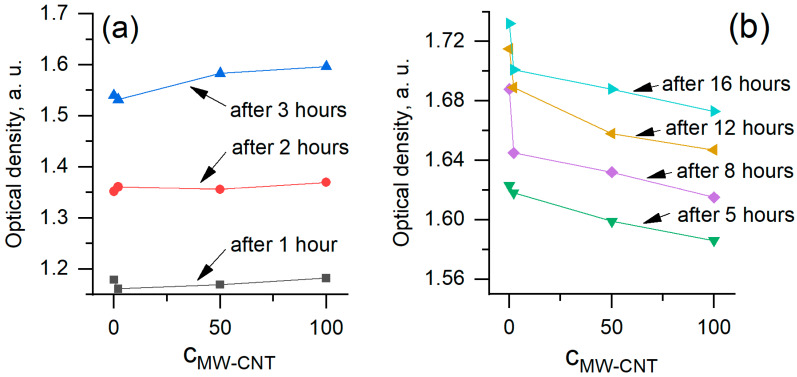
Dependence of optical density on MW-CNTs concentration at a different time: (**a**) 1–3 h; (**b**) 5–16 h.

In order to analyse the data in more detail, we plotted the same data as optical density vs. MW-CNTs dependencies (Figure 7). Data of experiments performed at 1–3 h (Figure 6a) were plotted separately after 5–16 h (Figure 6b) to show different MW-CNTs effect on yeast cells. We obtained that at the very beginning of the treatment, within 1 to 3 h, MW-CNTs do not affect yeast cells or the effect is very small. The optical density increased with increasing MW-CNTs concentration. Measurement after 5 h show a decrease in optical density, revealing the toxic effect of MW-CNTs. However, the optical density at 0 µg/mL and 2 µg/mL MW-CNTs are practically the same, hence toxic effect after 5 h is observed at the higher MW-CNTs concentrations. The highest difference at 0 µg/mL and 2 µg/mL MW-CNTs is seen after 8 h of the experiment. This may be a critical time, at which the reaction of yeast to the MW-CNTs becomes sufficiently high to be registered. Data from all measurement period clearly reveal the toxic effect of MW-CNTs (Figure 7b), while the dependence of optical density on time (Figure 6) do not show this effect. Measurements of optical density, performed at 20, 22, and 24 h, do not show reliable dependencies, and they are not shown here. These time points also are not in the accordance with the model (Figure 6), especially at the higher MW-CNTs concentrations.

**Figure 8:**
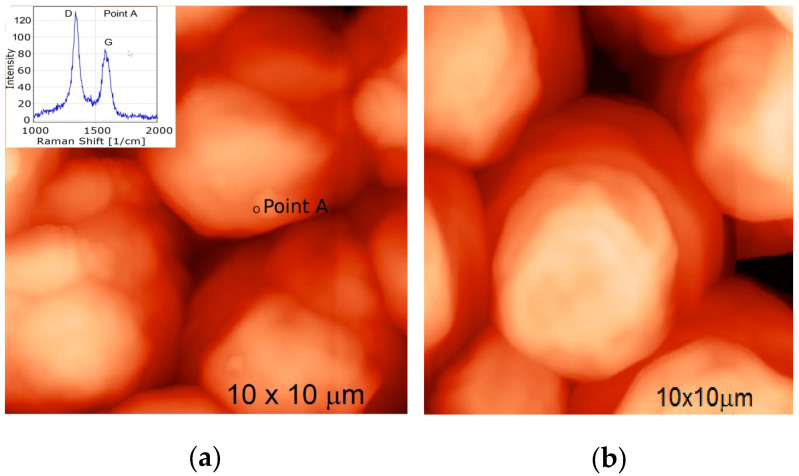
AFM image of yeast cells: (**a**) AFM image of yeast cells and Raman spectra of MW-CNTs at the point of MW-CNTs at the point A (insert), (**b**) AFM image of yeast cells without MW-CNTs.

The investigation of the interaction of MW-CNTs with the yeast *Saccharomyces cerevisiae* cells was done using integrated AFM and confocal Raman spectroscopy (Spectra, NT-MDT). The AFM image of the yeast cells is presented in Figure 8b, and confocal Raman spectra collected from the selected points on the cells surface is presented in inset of Figure 8a. The Raman spectra of biological components from the yeast cell surface was subtracted as background during the Raman spectra measurement. The collected MW-CNTs Raman spectra from the cells surface confirm the MW-CNTs interaction with the yeast cells.

### 3.3. Electrochemical Analysis and Biofuel Cell Development

**Figure 9:**
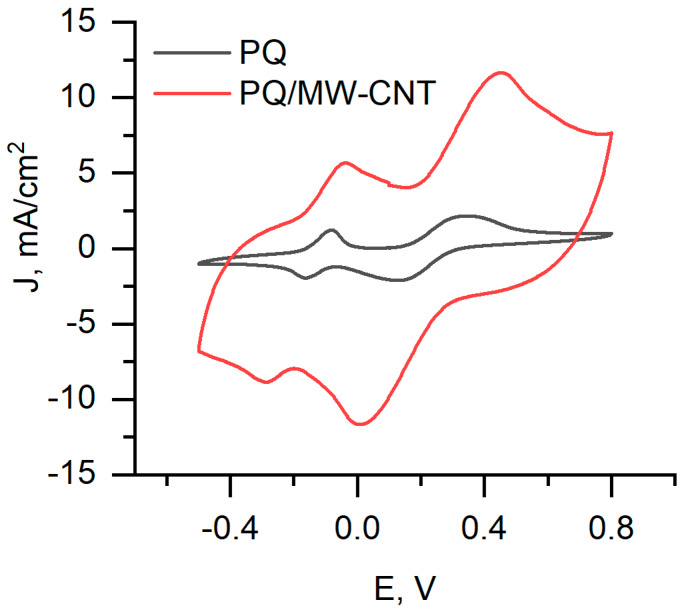
Cyclic voltammograms of PQ and PQ/MW-CNT modified biofuel cell anode (graphite electrode), immersed in a phosphate buffer solution with 10 mM K_3_[Fe(CN)_6_], 70 mM glucose, and 3 mg/mL yeast cells.

In this research, we used the two-redox mediators-based system, which has been described in previous our research [31]. One redox mediator PQ was used for modification of graphite electrode, and another one—K_3_[Fe(CN)_6_]—was added in the solution. PQ has the ability to cross the cell’s membrane and achieve redox centres of enzymes located in the cytoplasm of cells. K_3_[Fe(CN)_6_] takes electrons from PQ and passes them to the electrode. We registered cyclic voltammograms using PQ, and PQ/MW-CNT modified graphite electrode (Figure 9). Four peaks were observed in both cyclic voltammograms, PQ oxidation/reduction peaks (two on the left) and [Fe(CN)_6_]^3–/4–^ oxidation/reduction peaks. Comparing measurement using PQ and PQ/MW-CNT graphite electrodes, we can conclude that using of MW-CNTs allows us to increase the current for both PQ and [Fe(CN)_6_]^3–/4–^ oxidation/reduction processes.

**Figure 10:**
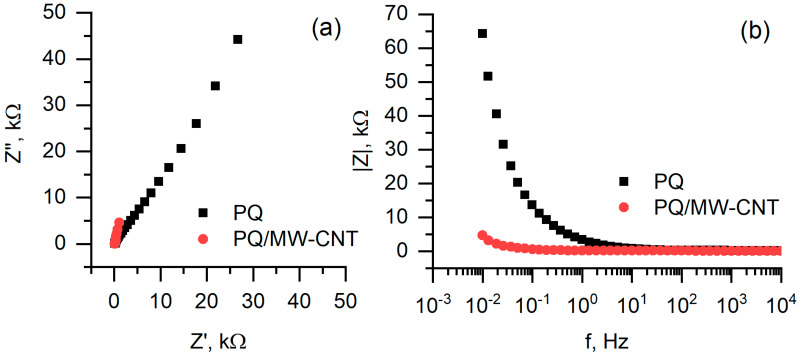
Electrochemical impedance spectroscopy based measurements: (**a**) Nyquist plot, (**b**) Bode plot. Measurements conditions are the same as in Figure 9.

Electrochemical impedance spectroscopy based measurements represent the resistivity of the layers, formed at anode surface (Figure 10). In case when PQ-modified anode was used, charge transfer resistance is much higher comparing to hat of biofuel cell based on PQ/MW-CNT electrode. This effect is well observed in both Nyquist (Figure 10a) and Bode plots (Figure 10b).

**Figure 11:**
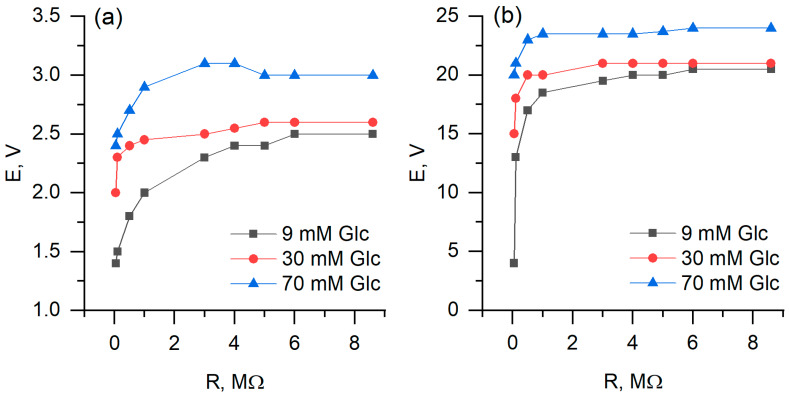
Generated potential dependence on applied load, using (**a**) PQ- and (**b**) PQ/MW-CNT-modified graphite electrode as an anode.

**Figure 12:**
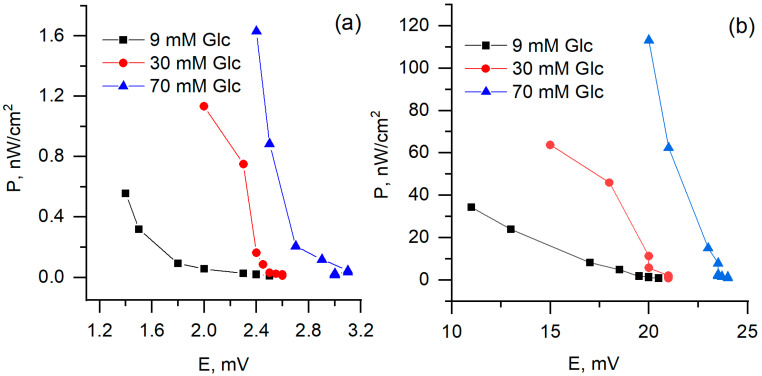
Generated power dependence on potential, (**a**) PQ- and (**b**) PQ/MW-CNT-modified graphite electrode as an anode.

During measurements of biofuel cell performance, the two-electrode-based electrochemical cell was used, with one PQ or PQ/MW-CNT modified graphite based electrode and another—platinum-based electrode (Figure 10). It was determined that biofuel cell, in which PQ/MW-CNT modified electrode was applied, generated 8 times higher potential at the load of 8.6 MΩ, and 70 mM glucose concentration: potential, measured with PQ-modified electrode, was 3 mV while using PQ/MW-CNT electrode 24 mV potential was observed (Figure 11a). At the same conditions calculated maximal power for PQ/MW-CNT-electrode was 113 nW/cm^2^, while for PQ-electrode maximal power was just 1.63 nW/cm^2^, i.e., for PQ/MW-CNT power was 69 times higher (Figure 12). Therefore, it can be concluded that MW-CNTs has a good potential to be used in microbial fuel cells.

## 4. Conclusions and Future Trends

It was determined that MW-CNTs have an observable effect on the development of the yeast population upon the incubation of the yeast cells treated with the nanotubes for 24 h. It was determined that at lowest here investigated concentration (2 µg/mL) of MW-CNTs and at rather short-lasting exposure of MW-CNTs do not significantly affect the viability and other properties of yeast cells. At higher concentrations and after longer exposure, the increase of optical density vs. MW-CNTs concentration is observed. A plot of optical density vs. incubation time dependencies, show some differences between low (0 and 2 µg/mL) and high (50 and 100 µg/mL) MW-CNTs concentrations. The steady-state conditions were observed faster for high MW-CNTs concentrations. Also, calculated relative growth and relative death rates show the difference between results registered after incubation in low and high MW-CNTs concentrations containing solutions: both rates were two-times higher for higher concentrations of MW-CNTs. The results, obtained from electrochemical measurements, enables to state that MW-CNTs are a very good candidates for the development of microbial fuel cells, because the application of MW-CNTs in anode of biofuel cell increased generated power by 69 times and generated voltage by 8 times. Therefore, we predict that MW-CNTs can be applied for the modification of the yeast cells in order to improve electrical charge transfer through the yeast cell membrane and/or cell wall. Further, our investigations in this research area will be related to the development of a strategy of how MW-CNTs can be inserted within the yeast cell wall and membrane in order to apply such modified cells in the design of microbial fuel cell.

## Figures and Tables

**Table 1:** Parameters of the model.

	Relative Growth Rate (*α*), h^−1^	Relative Death Rate (*µ*), h^−1^	Consumption of Non-Saccharide Resources (*ζ*)	Initial Population Size x(0) *
Unaffected cells	0.215	0.0207	5	1
2 µg/mL MW-CNTs	0.219	0.022	5	1
50 µg/mL MW-CNTs	0.46	0.041	4.8	0.8
100 µg/mL MW-CNTs	0.46	0.041	4.8	0.8

* z(0) = 1 was used in all simulations.
